# Bis(4-amino­pyridinium) bis(hydrogen oxalate) monohydrate

**DOI:** 10.1107/S1600536809007247

**Published:** 2009-03-14

**Authors:** Hoong-Kun Fun, Jain John, Samuel Robinson Jebas, T Balasubramanian

**Affiliations:** a X-ray Crystallography Unit, School of Physics, Universiti Sains Malaysia, 11800 USM, Penang, Malaysia; b Department of Physics, National Institute of Technology, Tiruchirappalli 620015, India

## Abstract

In the title compound, 2C_5_H_7_N_2_^+^·2C_2_HO_4_^−^·H_2_O, the asymmetric unit consists of an amino­pyridinium cation, an oxalic actetate anion and a half-molecule of water, which lies on a two-fold rotation axis. The crystal packing is consolidated by inter­molecular O—H⋯O, N—H⋯O and C—H⋯O hydrogen bonds. The mol­ecules are linked into an infinite one dimensional chain along [010].

## Related literature

For the biological activity of 4-amino­pyridine, see: Judge & Bever (2006 ▶); Schwid *et al.* (1997 ▶); Strupp *et al.* (2004 ▶). For the structure of oxalic acid, see: Derissen & Smith (1974 ▶). For related structures, see: Anderson *et al.* (2005 ▶); Bhattacharya *et al.* (1994 ▶); Chao & Schempp (1977 ▶); Karle *et al.* (2003 ▶). For stability of the temperature controller, see: Cosier & Glazer (1986 ▶). 
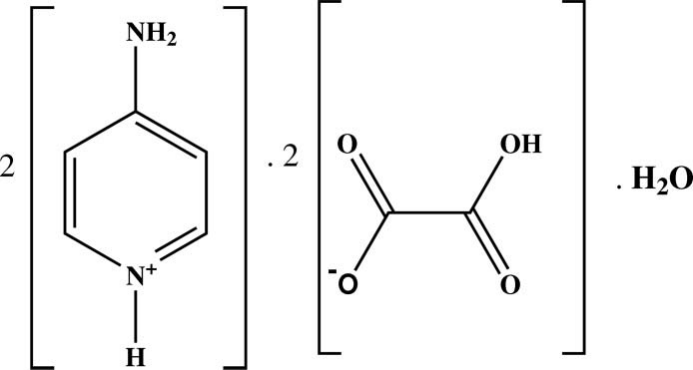


## Experimental

### 

#### Crystal data

2C_5_H_7_N_2_^+^·2C_2_HO_4_^−^·H_2_O
*M* *_r_* = 386.32
Monoclinic, 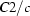

*a* = 15.6429 (6) Å
*b* = 5.6929 (2) Å
*c* = 19.9091 (7) Å
β = 105.617 (2)°
*V* = 1707.52 (11) Å^3^
*Z* = 4
Mo *K*α radiation
μ = 0.13 mm^−1^
*T* = 100 K
0.49 × 0.34 × 0.11 mm

#### Data collection

Bruker SMART APEXII CCD area-detector diffractometer
Absorption correction: multi-scan (*SADABS*; Bruker, 2005 ▶) *T*_min_ = 0.906, *T*_max_ = 0.986
12438 measured reflections
2467 independent reflections
2159 reflections with *I* > 2σ(*I*)
*R*_int_ = 0.027

#### Refinement

*R*[*F* ^2^ > 2σ(*F* ^2^)] = 0.034
*wR*(*F* ^2^) = 0.096
*S* = 1.03
2467 reflections
159 parameters
All H-atom parameters refined
Δρ_max_ = 0.35 e Å^−3^
Δρ_min_ = −0.24 e Å^−3^

### 

Data collection: *APEX2* (Bruker, 2005 ▶); cell refinement: *SAINT* (Bruker, 2005 ▶); data reduction: *SAINT*; program(s) used to solve structure: *SHELXTL* (Sheldrick, 2008 ▶); program(s) used to refine structure: *SHELXTL*; molecular graphics: *SHELXTL*; software used to prepare material for publication: *SHELXTL* and *PLATON* (Spek, 2009 ▶).

## Supplementary Material

Crystal structure: contains datablocks global, I. DOI: 10.1107/S1600536809007247/at2732sup1.cif

Structure factors: contains datablocks I. DOI: 10.1107/S1600536809007247/at2732Isup2.hkl

Additional supplementary materials:  crystallographic information; 3D view; checkCIF report

## Figures and Tables

**Table 1:** Hydrogen-bond geometry (Å, °)

*D*—H⋯*A*	*D*—H	H⋯*A*	*D*⋯*A*	*D*—H⋯*A*
O1*W*—H2*W*1⋯O4^i^	0.874 (18)	1.895 (18)	2.7676 (9)	175.0 (18)
N2—H1*N*2⋯O4^ii^	0.877 (15)	1.983 (16)	2.8556 (11)	173.4 (14)
N2—H2*N*2⋯O1*W*^iii^	0.890 (16)	1.993 (15)	2.8620 (12)	164.8 (13)
N1—H1*N*1⋯O3^iv^	0.863 (17)	2.100 (17)	2.8645 (11)	147.3 (15)
N1—H1*N*1⋯O2^iv^	0.863 (17)	2.218 (17)	2.8818 (11)	133.6 (15)
O1—H1*O*1⋯O3^v^	1.00 (2)	1.60 (2)	2.5916 (10)	177.6 (18)
C5—H5⋯O2^vi^	0.951 (13)	2.361 (14)	3.1585 (12)	141.2 (11)

Symmetry codes: (i) 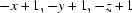; (ii) 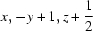; (iii) 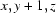; (iv) 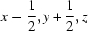; (v) 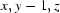; (vi) 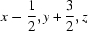.

**Fig. 1.**
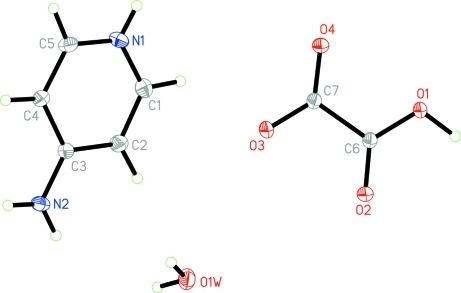
The molecular structure of the title compound, showing 50% probability displacement ellipsoids and the atom numbering scheme.

**Fig. 2.**
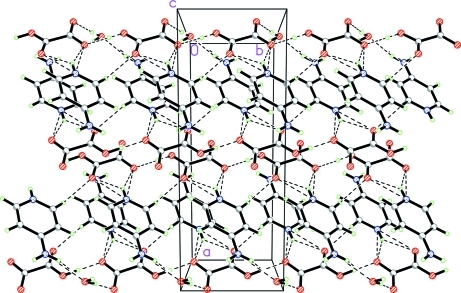
The crystal packing of the title compound, viewed down the c axis. Dashed lines indicate the hydrogen bonding.

## supplementary crystallographic information

### Comment

4-Aminopyridine (Fampridine) is used clinically in Lambert-Eaton myasthenic syndrome and multiple sclerosis because by blocking potassium channels it prolongs action potentials thereby increasing transmitter release at the neuromuscular junction (Judge & Bever, 2006; Schwid *et al.*, 1997; Strupp *et al.*, 2004). The structure of 4-aminopyridine has already been reported (Chao & Schempp, 1977). Redetermination of the structure of 4-aminopyridine has been reported (Anderson *et al.*, 2005). The crystal structure of oxalic acid monohydrate has been reported (Derissen & Smith, 1974). As an extension of our systematic study of the hydrogen bonding patterns of 4-aminopyridine with carboxylic acids, the title compound (I) has been synthesized and the crystal structure determined.

The asymmetric unit of (I) (Fig. 1) contains one molecule of 4-aminopyridine cation, one molecule of oxalate anion and half-a-molecule of water. A proton transfer from the carboxyl group of oxalic acid to atom N1 of 4-aminopyridine resulted in the formation of salts. This protonation lead to the widening of C1–N1–C5 angle of the pyridine ring to 121.0 (8)°, compared to 115.25 (13)° in the unprotonated 4-aminopyridine (Anderson *et al.*, 2005). This type of protonation is observed in various 4-aminopyridine acid complexes (Bhattacharya *et al.*, 1994; Karle *et al.*, 2003). The bond lengths and bond angles of the 4-aminopyridine are comparable to the values reported earlier for 4-aminopyridine (Chao & Schempp, 1977; Anderson *et al.*, 2005). The 4-aminopyridine ring is essentially planar with the maximum deviation from planarity being 0.0075 (9)Å for atom N1. The bond lengths and bond angles of the oxalate are comparable to the values reported for oxalic acid (Derissen & Smith, 1974).

The crystal packing is consolidated by intermolecular O—H···O, N—H···O and C—H···O hydrogen bonds (Table 1). Intermolecular short contacts of O—O = 2.5916 (10)^i^ to 2.7074 (10)Å and N—O = 2.8557 (11)^ii^ to 2.8646 (11)^iii^Å are observed [symmetry codes: (i) *x*,-1 + *y*,*z*; (ii) *x*,1 - *y*,1/2 + *z*; (iii) -1/2 + *x*,1/2 + *y*,*z*]. The molecules are linked into a 3-D network (Fig. 2).

### Experimental

Equimolar quantities of 4-aminopyridine (0.094 g, 1 mmol) and oxalic acid (0.090 g, 1 mmol) were dissolved in 25 ml water. The solution was refluxed at 323 K for 12 h. Colourless crystals were harvested after two months of solvent evaporation.

### Refinement

All the hydrogen atoms were located from the Fourier map and were allowed to refine freely.

### Crystal data

**Table:**

2C_5_H_7_N_2_^+^·2C_2_HO_4_^−^·H_2_O	*F*(000) = 808
*M**_r_* = 386.32	*D*_x_ = 1.503 Mg m^−^^3^
Monoclinic, *C*2/*c*	Mo *K*α radiation, λ = 0.71073 Å
Hall symbol: -C 2yc	Cell parameters from 7009 reflections
*a* = 15.6429 (6) Å	θ = 2.7–38.8°
*b* = 5.6929 (2) Å	µ = 0.13 mm^−^^1^
*c* = 19.9091 (7) Å	*T* = 100 K
β = 105.617 (2)°	Plate, colourless
*V* = 1707.52 (11) Å^3^	0.49 × 0.34 × 0.11 mm
*Z* = 4	

### Data collection

**Table:**

Bruker SMART APEXII CCD area-detector diffractometer	2467 independent reflections
Radiation source: fine-focus sealed tube	2159 reflections with *I* > 2σ(*I*)
graphite	*R*_int_ = 0.027
φ and ω scans	θ_max_ = 30.0°, θ_min_ = 2.1°
Absorption correction: multi-scan (*SADABS*; Bruker, 2005)	*h* = −22→21
*T*_min_ = 0.906, *T*_max_ = 0.986	*k* = −7→7
12438 measured reflections	*l* = −27→28

### Refinement

**Table:**

Refinement on *F*^2^	Primary atom site location: structure-invariant direct methods
Least-squares matrix: full	Secondary atom site location: difference Fourier map
*R*[*F*^2^ > 2σ(*F*^2^)] = 0.034	Hydrogen site location: inferred from neighbouring sites
*wR*(*F*^2^) = 0.096	All H-atom parameters refined
*S* = 1.03	*w* = 1/[σ^2^(*F*_o_^2^) + (0.0564*P*)^2^ + 0.8323*P*] where *P* = (*F*_o_^2^ + 2*F*_c_^2^)/3
2467 reflections	(Δ/σ)_max_ < 0.001
159 parameters	Δρ_max_ = 0.35 e Å^−^^3^
0 restraints	Δρ_min_ = −0.24 e Å^−^^3^

### Special details

**Table:**

Experimental. The crystal was placed in the cold stream of an Oxford Cyrosystems Cobra open-flow nitrogen cryostat (Cosier & Glazer, 1986) operating at 100.0 (1) K.
Geometry. All e.s.d.'s (except the e.s.d. in the dihedral angle between two l.s. planes) are estimated using the full covariance matrix. The cell e.s.d.'s are taken into account individually in the estimation of e.s.d.'s in distances, angles and torsion angles; correlations between e.s.d.'s in cell parameters are only used when they are defined by crystal symmetry. An approximate (isotropic) treatment of cell e.s.d.'s is used for estimating e.s.d.'s involving l.s. planes.
Refinement. Refinement of *F*^2^ against ALL reflections. The weighted *R*-factor *wR* and goodness of fit *S* are based on *F*^2^, conventional *R*-factors *R* are based on *F*, with *F* set to zero for negative *F*^2^. The threshold expression of *F*^2^ > σ(*F*^2^) is used only for calculating *R*-factors(gt) *etc*. and is not relevant to the choice of reflections for refinement. *R*-factors based on *F*^2^ are statistically about twice as large as those based on *F*, and *R*- factors based on ALL data will be even larger.

### Fractional atomic coordinates and isotropic or equivalent isotropic displacement parameters (Å^2^)

**Table:**

	*x*	*y*	*z*	*U*_iso_*/*U*_eq_	
O1	0.50462 (4)	−0.16951 (12)	0.39445 (3)	0.01643 (15)	
O2	0.59652 (5)	−0.02380 (12)	0.49260 (3)	0.01700 (16)	
O3	0.55682 (5)	0.41624 (12)	0.44228 (4)	0.02023 (17)	
O4	0.45356 (5)	0.26647 (12)	0.35138 (3)	0.01903 (16)	
N1	0.18206 (5)	0.86694 (16)	0.57611 (4)	0.01960 (18)	
N2	0.39056 (6)	1.06075 (16)	0.74035 (4)	0.01829 (17)	
C1	0.20886 (7)	0.72101 (18)	0.63132 (5)	0.0199 (2)	
C2	0.27784 (6)	0.77998 (18)	0.68707 (5)	0.01844 (19)	
C3	0.32276 (6)	0.99712 (16)	0.68737 (5)	0.01467 (18)	
C4	0.29134 (6)	1.14587 (17)	0.62850 (5)	0.01667 (19)	
C5	0.22175 (6)	1.07631 (18)	0.57489 (5)	0.0188 (2)	
C6	0.54297 (6)	0.00122 (16)	0.43617 (4)	0.01357 (17)	
C7	0.51411 (6)	0.25017 (16)	0.40655 (5)	0.01471 (18)	
O1W	0.5000	0.46896 (19)	0.7500	0.0215 (2)	
H2W1	0.5135 (12)	0.560 (3)	0.7190 (9)	0.048 (5)*	
H1	0.1765 (9)	0.574 (3)	0.6285 (7)	0.028 (3)*	
H2	0.2966 (9)	0.673 (3)	0.7268 (8)	0.029 (4)*	
H4	0.3195 (9)	1.295 (3)	0.6250 (7)	0.023 (3)*	
H5	0.1983 (9)	1.169 (2)	0.5344 (7)	0.021 (3)*	
H1N2	0.4086 (10)	0.968 (3)	0.7766 (8)	0.028 (4)*	
H2N2	0.4168 (9)	1.197 (3)	0.7367 (7)	0.025 (3)*	
H1N1	0.1413 (11)	0.823 (3)	0.5397 (9)	0.038 (4)*	
H1O1	0.5253 (12)	−0.327 (4)	0.4140 (9)	0.054 (5)*	

### Atomic displacement parameters (Å^2^)

**Table:**

	*U*^11^	*U*^22^	*U*^33^	*U*^12^	*U*^13^	*U*^23^
O1	0.0207 (3)	0.0102 (3)	0.0151 (3)	0.0000 (2)	−0.0007 (2)	−0.0013 (2)
O2	0.0197 (3)	0.0137 (3)	0.0141 (3)	0.0009 (2)	−0.0014 (3)	0.0004 (2)
O3	0.0259 (4)	0.0109 (3)	0.0186 (3)	−0.0014 (2)	−0.0033 (3)	−0.0011 (2)
O4	0.0249 (4)	0.0141 (3)	0.0136 (3)	0.0010 (2)	−0.0027 (3)	0.0007 (2)
N1	0.0176 (4)	0.0215 (4)	0.0160 (4)	−0.0005 (3)	−0.0017 (3)	−0.0030 (3)
N2	0.0178 (4)	0.0182 (4)	0.0152 (4)	−0.0017 (3)	−0.0019 (3)	0.0011 (3)
C1	0.0196 (4)	0.0165 (5)	0.0221 (5)	−0.0023 (3)	0.0031 (4)	−0.0012 (3)
C2	0.0192 (4)	0.0166 (4)	0.0176 (4)	−0.0011 (3)	0.0017 (3)	0.0028 (3)
C3	0.0149 (4)	0.0143 (4)	0.0140 (4)	0.0012 (3)	0.0022 (3)	−0.0006 (3)
C4	0.0177 (4)	0.0143 (4)	0.0165 (4)	0.0008 (3)	0.0019 (3)	0.0017 (3)
C5	0.0182 (4)	0.0205 (5)	0.0152 (4)	0.0032 (3)	0.0002 (3)	0.0025 (3)
C6	0.0158 (4)	0.0112 (4)	0.0133 (4)	−0.0001 (3)	0.0032 (3)	−0.0002 (3)
C7	0.0188 (4)	0.0112 (4)	0.0132 (4)	0.0003 (3)	0.0027 (3)	0.0002 (3)
O1W	0.0307 (6)	0.0146 (5)	0.0188 (5)	0.000	0.0061 (4)	0.000

### Geometric parameters (Å, °)

**Table:**

O1—C6	1.3139 (11)	C1—C2	1.3654 (13)
O1—H1O1	1.00 (2)	C1—H1	0.973 (15)
O2—C6	1.2155 (11)	C2—C3	1.4211 (13)
O3—C7	1.2611 (11)	C2—H2	0.979 (15)
O4—C7	1.2458 (11)	C3—C4	1.4221 (12)
N1—C5	1.3471 (14)	C4—C5	1.3621 (13)
N1—C1	1.3514 (13)	C4—H4	0.966 (15)
N1—H1N1	0.863 (17)	C5—H5	0.951 (13)
N2—C3	1.3291 (12)	C6—C7	1.5547 (13)
N2—H1N2	0.877 (15)	O1W—H2W1	0.874 (18)
N2—H2N2	0.890 (16)		
			
C6—O1—H1O1	111.9 (11)	N2—C3—C4	121.12 (9)
C5—N1—C1	121.00 (8)	C2—C3—C4	116.98 (8)
C5—N1—H1N1	118.7 (11)	C5—C4—C3	119.91 (9)
C1—N1—H1N1	120.2 (11)	C5—C4—H4	118.8 (8)
C3—N2—H1N2	120.1 (10)	C3—C4—H4	121.3 (8)
C3—N2—H2N2	117.4 (9)	N1—C5—C4	121.21 (9)
H1N2—N2—H2N2	122.5 (13)	N1—C5—H5	115.6 (8)
N1—C1—C2	120.95 (9)	C4—C5—H5	123.2 (9)
N1—C1—H1	116.1 (8)	O2—C6—O1	125.55 (8)
C2—C1—H1	123.0 (8)	O2—C6—C7	121.00 (8)
C1—C2—C3	119.93 (9)	O1—C6—C7	113.45 (7)
C1—C2—H2	120.2 (9)	O4—C7—O3	127.09 (8)
C3—C2—H2	119.9 (9)	O4—C7—C6	118.46 (8)
N2—C3—C2	121.90 (9)	O3—C7—C6	114.44 (8)
			
C5—N1—C1—C2	−0.98 (15)	C1—N1—C5—C4	1.41 (15)
N1—C1—C2—C3	−0.29 (15)	C3—C4—C5—N1	−0.56 (15)
C1—C2—C3—N2	−179.57 (9)	O2—C6—C7—O4	−173.95 (8)
C1—C2—C3—C4	1.08 (14)	O1—C6—C7—O4	6.47 (12)
N2—C3—C4—C5	179.98 (9)	O2—C6—C7—O3	6.58 (13)
C2—C3—C4—C5	−0.67 (14)	O1—C6—C7—O3	−173.00 (8)

### Hydrogen-bond geometry (Å, °)

**Table:**

*D*—H···*A*	*D*—H	H···*A*	*D*···*A*	*D*—H···*A*
O1W—H2W1···O4^i^	0.874 (18)	1.895 (18)	2.7676 (9)	175.0 (18)
N2—H1N2···O4^ii^	0.877 (15)	1.983 (16)	2.8556 (11)	173.4 (14)
N2—H2N2···O1W^iii^	0.890 (16)	1.993 (15)	2.8620 (12)	164.8 (13)
N1—H1N1···O3^iv^	0.863 (17)	2.100 (17)	2.8645 (11)	147.3 (15)
N1—H1N1···O2^iv^	0.863 (17)	2.218 (17)	2.8818 (11)	133.6 (15)
O1—H1O1···O3^v^	1.00 (2)	1.60 (2)	2.5916 (10)	177.6 (18)
C5—H5···O2^vi^	0.951 (13)	2.361 (14)	3.1585 (12)	141.2 (11)

Symmetry codes: (i) −*x*+1, −*y*+1, −*z*+1; (ii) *x*, −*y*+1, *z*+1/2; (iii) *x*, *y*+1, *z*; (iv) *x*−1/2, *y*+1/2, *z*; (v) *x*, *y*−1, *z*; (vi) *x*−1/2, *y*+3/2, *z*.
